# Leukemic transformation driven by an *ASXL1* mutation after a *JAK2V617F*-positive primary myelofibrosis: clonal evolution and hierarchy revealed by next-generation sequencing

**DOI:** 10.1186/1756-8722-6-68

**Published:** 2013-09-08

**Authors:** Francisca Ferrer-Marín, Beatriz Bellosillo, Luz Martínez-Avilés, Gloria Soler, Pablo Carbonell, Ginés Luengo-Gil, Eva Caparrós, José M Torregrosa, Carlos Besses, Vicente Vicente

**Affiliations:** 1 Hematology and Medical Oncology Unit, Hospital Universitario Morales-Meseguer, Centro Regional de Hemodonación, C/Ronda de Garay, sn. 3003, Murcia, Spain; 2 Department of Pathology, Hospital del Mar-IMIM, Barcelona, Spain; 3 Molecular Genetics Unit, Hospital Universitario Virgen de la Arrixaca, Murcia, Spain; 4 Department of Hematology, Hospital del Mar-IMIM, Barcelona, Spain

**Keywords:** Myelofibrosis, *ASXL1*, Gene mutations, Acute myeloid leukemia, Clonality, Next-generation sequencing

## Abstract

We have characterized the molecular changes underlying the transformation of a *JAK2V617F*^+^-myelofibrosis with trisomy 8, into a *JAK2V617F*-negative leukemia. Leukemic clone did not carry *JAK2V617F* mutation, but showed *ASXL1* mutation (R693X). This mutation was identified in a low percentage at diagnosis by next-generation sequencing. Using this technology in serial specimens during the follow-up, we observed a progressive expansion of the *ASXL1*-mutated minor clone, whereas the *JAK2V617F*^+^-clone carrying trisomy 8 decreased. Hematologic progression occurred simultaneously with an *ASXL1*-R693X-negative lung-cancer. This is the first report showing a clear association between the expansion of an *ASXL1*-mutated clone and the leukemic transformation of myelofibrosis.

## Letters to the editor

It is known that patients with *JAK2V617F*^*+*^ myeloproliferative neoplasms (MPNs) can progress to a *JAK2V617F¯* acute myeloid leukemia (AML) [1-3]. These two phases of the disease may represent two different clones [1], however, the time-dependent clonal hierarchy is just beginning to be elucidated [4,5].

*ASXL1* is the second most frequently mutated gene after *JAK2* (~34.5%) in myelofibrosis (MF) [6]. *ASXL1* mutations are also found in solid neoplasms and all types of myeloid malignancies [7]. They are associated with aggressive disease [8] but their role in leukemic transformation remains controversial. *ASXL1* mutations correlated with progression to blast-state in myelodysplastic syndromes and chronic myelomonocytic leukaemia [9,10] while in MF, they are detectable in most patients at diagnosis [11], and they are present in chronic- and blast-phases [2] with the same prevalence [6]. These findings suggest that *ASXL1* mutations play a crucial role in the pathogenesis of MF [6,11] but they do not directly cause a leukemic phenotype [2,6]. We here characterize the molecular changes associated to the leukemic transformation of a patient with primary-MF (PMF) using next-generation sequencing (NGS). By the time of the hematologic progression, the patient also developed a lung adenocarcinoma. The relationship between the clonal hierarchy and phenotype disease over time are discussed.

## Case presentation

**Figure 1:**
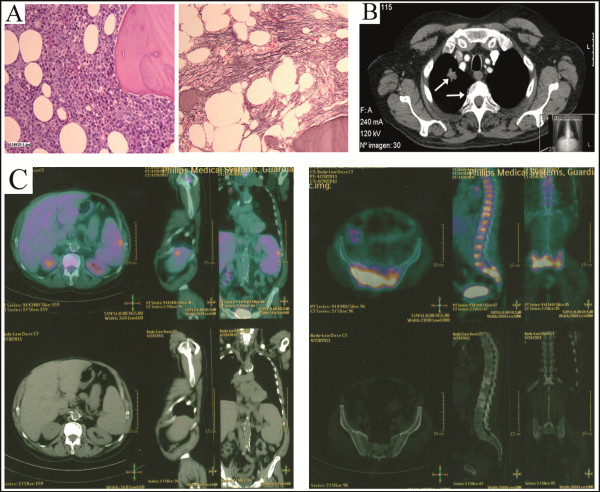
**Histologic and radiologic studies at the time of leukemic transformation. (A)** BM biopsy showing immature myeloid proliferation without segmentation (blasts >20%) and dysplastic megakaryocytes. With silver staining (right panel), marked reticulin fibrosis associated with osteoesclerosis was also shown. **(B)** Chest CT scan showing an increase in bone density of the vertebral bodies and a right lung nodule of 2 × 2 cm in size with speculated edges. **(C)** Positron-emission tomography with CT showing a markedly increased of ^18^ F-FDG uptake in the BM of the vertebral bodies; sacrum; extremity bones (specially left humerus); and focal in the spleen.

A 62-year-old male with 126 pack-year smoking history, cardiomyopathy and chronic-pulmonary disease was referred to our Department in May’ 2007 for evaluation of anemia, splenomegaly and fever. Following a peripheral blood (PB) and a bone marrow (BM) examination, a diagnosis of PMF according to WHO criteria was made, IPSS intermediate-2 [12]. Genetic analysis revealed a trisomy 8 in all 18 metaphases analyzed and the *JAK2V617F* mutation. With hydroxyurea, the patient achieved a complete resolution of fever and splenomegaly, and hemoglobin normalization. Thirty months later, he started with lumbar pain and leukocytosis (12.9 × 10^9^/L). A new BM biopsy showed severe fibrosis with osteosclerosis. Due to the patients’s comorbidities, allogeneic transplant was not possible and hydroxyurea was continued. In December’ 2010, the patient developed fever, splenomegaly, marked leukocytosis (81.3 × 10^9^/L) and required red-cell transfusions. A new BM biopsy confirmed leukemic transformation (Figure 1A) but fluorescence *in situ* hybridization (FISH) analysis discarded trisomy 8. Hydroxyurea was increased achieving a good control of leukocyte count. Few weeks later, he began with back and thoracic pain. A computed tomography (CT) scan revealed a speculated right lung nodule (Figure 1B). Biopsy confirmed non–small-cell lung cancer (NSCLC) (T1N0MX) and a PET-CT for staging was made (Figure 1C). Despite starting treatment for AML with thioguanine, the patient died because of acute congestive heart and liver failure. A post-mortem liver biopsy uncovered metastatic infiltration by NSCLC.

## Discussion

**Figure 2:**
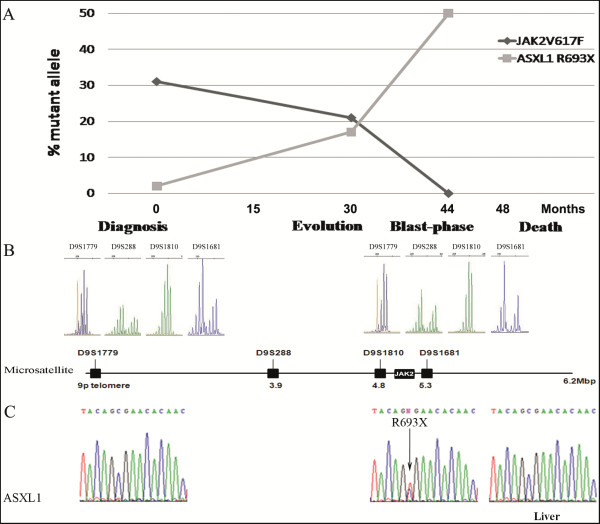
**Clonal progression from diagnosis of PMF to leukemic transformation and death. (A)** Dynamic changes in the size of the two mutated clones, *JAK2V617F* and *ASXL1*-R693X, in three time points during follow up: at the time of presentation of PMF, during the evolution and in blast-phase. **(B)** Microsatellite analysis on PB granulocytes at diagnosis (left panel) and blast-phase of MF (right panel) for 4 markers on chromosome 9p flanking JAK2. The positions of microsatellite markers used to identify the common 9pLOH region are shown as vertical lines **(C)** ASXL1 sequencing in paired samples of cDNA from PB granulocytes showing one nonsense mutation (R693X) in the *ASXL1* gene at blast-phase, but not at diagnosis (left panel). This mutation was not detected in the hepatic metastatic tissue of lung cancer (right panel).

To further investigate the molecular mechanisms underlying neoplastic progression in this case, we performed *JAK2V617F* allele burden in PB granulocytes obtained at three time points, by allele-specific qRT-PCR [13]. *JAK2V617F* allele percentage decreased from 30% at diagnosis to undetectable level at blast-phase with an intermediate value of 20% (Figure 2A). A clonal analysis with microsatellites for 4 markers on chromosome 9p flanking *JAK2,* on PB granulocytes taken at diagnosis and in the blast-phase, [14] did not show loss of heterozygosity on chromosome 9p (9pLOH), either at diagnosis (*JAK2V617F*^*+*^-clone) or at blast-stage (dominant *JAK2V617F¯*-clone) (Figure 2B), suggesting that at presentation, two independent clones were likely present in our patient. To address this question, sequencing of *ASXL1* (exon 12), *TET2* (all exons), *TP53* (exon 4–9), *IDH1* (R132), *IDH2* (R140, R172), *c-CBL* (exons 8–9) by Sanger and *SRSF2* (exon 1), *SF3B1* (exons 14–15) by NGS (454-GS Junior platform) were performed, in the same samples, as described [15,16]. At blast-phase, we identified an *ASXL1*-nonsense mutation (R693X), which was not present at diagnosis either by conventional sequencing (Figure 2C) or by high resolution melting analysis (not shown). However, by using NGS (sensibility 1%, [17]) we were able to detect the *ASXL1*-R693X mutation at diagnosis in a very low level (2%). Additionally, using NGS at two time-points during follow-up, we observed an expansion of the *ASXL1*-R693X subclone, with a maximum value of 50% at blast-phase (Figure 2A).

Since *ASXL1* has been involved in epithelial malignancy tumorigenesis [18] and cancer [7], we sequenced *ASXL1* gene in the hepatic metastatic tissue of lung cancer, but *ASXL1*-R693X mutation was not detected (Figure 2C), suggesting that at least three malignant clones might be present.

Overall, in this patient, at early disease, the PMF phenotype was driven mainly by a *JAK2V617F*^*+*^-dominant clone carrying trisomy 8. During the evolution this clone declined, whereas the *ASXL1*-mutated minor clone expanded, promoting the progression to leukemia. The reasons for these gradual shifts are unknown. Although hydroxyurea may induce a decrease in *JAK2V617F* allele burden in *JAK2V617F*^*+*^-MPNs [13,19], leukemic transformation in MF can occur without any prior therapy [20]. Furthermore, *ASXL1*-R693X mutation, as other mutations affecting genes with epigenetic role, likely favor the occurrence of secondary genetic events and, in association with other cooperating mutations, promotes blast-crisis [5].

The molecular mechanisms undergoing the myeloid leukemogenesis promoted by *ASXL1* have been recently reported [21], but in MF, the role of *ASXL1* mutations in leukemic transformation is still unclear [2,6,22]. By using NGS, we report, for the first time, an association between expansion of an *ASXL1*-mutated clone and MF progression to AML suggesting that in MF, as in other myeloid malignancies, *ASXL1* mutations play a role in leukemic transformation. Given the prevalence of *ASXL1* mutations in patients with MF, determination of *ASXL1* mutation status in these patients could help in the molecular disease monitoring.

## Consent

Written informed consent was obtained from the next of kin of the patient for publication of this Case report. A copy of the written consent is available for review by the Editor-in-Chief of this journal.

## Competing interests

The authors declare that they have no competing interests.

## Authors’ contributions

FFM provided clinical information, directed the study and wrote the manuscript. BB designed experiments, analyzed the sequencing data and contributed to the manuscript. LMA performed the molecular biology studies. GS performed cytogenetic and FISH analysis. PC performed the microsatellite analysis. EC and GLG contributed to perform molecular analysis experiments. JMT carried out acquisition of data’s patient. CB and VV supervised the study and were responsible for manuscript review. All authors have reviewed and approved the manuscript.
